# Neurological Severity Versus Biomarker Dynamics in Post-Stroke Dysphagia: A Dual-Pathway Model for Functional Recovery and Feeding Transition

**DOI:** 10.3390/jcm15082833

**Published:** 2026-04-08

**Authors:** Merve Savas, Senanur Kahraman Begen, Mehmet Serif Onen, Hafize Uzun

**Affiliations:** 1 Department of Speech and Language Therapy, Faculty of Health Sciences, Istanbul Atlas University, 34403 Istanbul, Türkiye; senanur.kahraman@atlas.edu.tr; 2 Department of Physical Medicine and Rehabilitation, Istanbul Atlas University Hospital, 34403 Istanbul, Türkiye; serif.onen@atlas.edu.tr; 3 Department of Medical Biochemistry, Faculty of Medicine, Istanbul Atlas University, 34403 Istanbul, Türkiye; hafize.uzun@atlas.edu.tr

**Keywords:** post-stroke dysphagia, enteral-to-oral feeding transition, prognostic nutritional index, systemic inflammation indices, swallowing function, aspiration

## Abstract

**Background:** Post-stroke dysphagia is a frequent complication associated with aspiration, malnutrition, and prolonged dependence on enteral feeding. Systemic inflammation and impaired nutritional status may adversely affect neuromuscular recovery; however, their relative and combined associations with swallowing recovery and transition from enteral to oral feeding remain insufficiently characterized. **Objective:** This study aimed to examine the independent associations of inflammatory and nutritional indices with swallowing function recovery and to evaluate their relationship with enteral-to-oral feeding transition in patients with post-stroke dysphagia. **Methods:** In this retrospective observational study, patients with dysphagia following ischemic stroke were evaluated before (T0) and after (T1) routine dysphagia rehabilitation. Inflammatory indices including the neutrophil-to-lymphocyte ratio (NLR), platelet-to-lymphocyte ratio (PLR), systemic immune–inflammation index (SII), systemic inflammation response index (SIRI), and pan-immune–inflammation value (PIV), as well as the prognostic nutritional index (PNI), were calculated at both time points. Changes in indices (Δ = T1 − T0) were analyzed in relation to changes in swallowing function assessed by the Functional Oral Intake Scale (FOIS) and the Penetration–Aspiration Scale (PAS). **Results:** Changes in PNI were independently associated with greater improvement in functional oral intake (ΔFOIS) and reductions in aspiration severity for both liquid and soft consistencies (ΔPAS; all *p* < 0.01). In contrast, changes in inflammatory indices (ΔSIRI, ΔSII, ΔPLR, and ΔPIV) were consistently associated with less favorable swallowing outcomes. In multivariable logistic regression analysis, baseline stroke severity (NIHSS) was the only independent determinant of transition from enteral to oral feeding (OR = 0.72, *p* = 0.002). The model demonstrated good discrimination (AUC = 0.81). **Conclusions:** Changes in nutritional status, as reflected by ΔPNI over time, were the biomarker most consistently associated with functional swallowing recovery and reduced aspiration severity in patients with post-stroke dysphagia. While inflammatory burden was associated with less favorable swallowing physiology, transition from enteral to oral feeding appeared to be primarily driven by neurological severity rather than inflammatory or nutritional indices alone. These findings may support the clinical value of monitoring nutritional reserve alongside inflammatory burden during dysphagia rehabilitation.

## 1. Introduction

Ischemic stroke is a pathobiological process that is not confined to focal brain injury but also triggers a pronounced systemic inflammatory response. The interaction between the inflammatory cascade initiated within ischemic tissue and the peripheral immune system leads to quantitative and functional alterations in neutrophil, monocyte, and lymphocyte populations, and these alterations have been reported to play a determining role in clinical course and functional outcomes [1]. Post-stroke systemic inflammation has been associated with complications and clinical outcomes, further suggesting that this biological response constitutes an important determinant of prognosis [1]. On the other hand, the impact of nutritional status on clinical outcomes has been robustly demonstrated [2]. In this context, it is increasingly recognized that the bidirectional interplay between inflammation and nutrition is critical in shaping disease trajectory and recovery.

In recent years, complete blood count-based composite indices have enabled a more comprehensive assessment of systemic inflammatory burden. Biomarkers such as the neutrophil-to-lymphocyte ratio (NLR) and platelet-to-lymphocyte ratio (PLR), which reflect distinct components of the immune response, have been associated with clinical outcomes. For instance, NLR has been shown to be significantly related to nutritional risk, with higher NLR values accompanying greater nutritional risk and less favorable clinical indicators [3]. Similarly, PLR has been reported to correlate with clinical scales reflecting nutritional status and to be potentially useful in predicting malnutrition [4]. Beyond these single ratios, the systemic inflammation response index (SIRI), which integrates neutrophil, monocyte, and lymphocyte counts, has been developed as a composite measure and has demonstrated independent prognostic value across clinical contexts [5]. In addition, the systemic immune–inflammation index (SII), which consolidates platelet, neutrophil, and lymphocyte components into a single metric, has been identified as a powerful marker for predicting disease course and outcomes [6]. Collectively, these findings indicate that inflammatory indices not only reflect immune response but may also indirectly capture aspects of nutritional reserve. A further marker that directly incorporates the nutritional dimension within this framework is the prognostic nutritional index (PNI), calculated based on serum albumin levels and peripheral lymphocyte count; PNI has been widely used in prognostic evaluation due to its significant associations with clinical outcomes [7]. As a more advanced extension of this approach, the pan-immune–inflammation value (PIV) is calculated as the ratio of neutrophil, platelet, and monocyte counts to lymphocyte count, thereby integrating components of inflammation, immune response, and nutritional status into a single composite variable. PIV has been strongly associated with disease severity and stage across different clinical settings; its negative correlation with albumin and its superior discriminative ability compared with single indices (e.g., NLR, SII) suggest that this parameter may provide a more comprehensive biological framework [8].

Dysphagia is a common post-stroke complication that can lead to serious outcomes such as aspiration pneumonia, malnutrition, and prolonged hospitalization. In clinical practice, swallowing function is primarily assessed using bedside screening tests and videofluoroscopic swallowing studies (VFSSs). While functional oral intake is quantitatively classified using the Functional Oral Intake Scale (FOIS), the severity of penetration and aspiration during VFSS is objectively graded using the Penetration–Aspiration Scale (PAS) [9,10]. Importantly, baseline stroke severity is a major determinant of dysphagia prognosis and post-stroke functional recovery and should therefore be accounted for when evaluating the independent effects of inflammatory and nutritional indices [11,12].

The influence of systemic inflammatory burden and nutritional reserve on swallowing recovery and transition from enteral to oral feeding has been addressed in only a limited number of studies. Therefore, composite inflammatory indices (NLR, PLR, SII, SIRI, PIV) and the prognostic nutritional index (PNI) in patients with post-stroke dysphagia may provide a pragmatic and objective approach to characterize clinical recovery. Nevertheless, for a functional clinical endpoint such as dysphagia, it has not yet been systematically compared which of these markers constitutes the strongest and most independent correlate of recovery.

The primary aim of this study is to comparatively evaluate the independent and relative associations of composite inflammatory indices (NLR, PLR, SII, SIRI, PIV) and the prognostic nutritional index (PNI) with transition from enteral to oral feeding and changes in swallowing function measured by FOIS and PAS. Primary hypothesis: Composite inflammatory indices (NLR, PLR, SII, SIRI, PIV) and the prognostic nutritional index (PNI) are independently associated with transition from enteral to oral feeding after adjustment for clinically relevant covariates, including baseline stroke severity and time since stroke. Secondary hypotheses: These indices are independently associated with improvement in swallowing outcomes, such that lower inflammatory burden and higher nutritional reserve are associated with greater improvement in FOIS and greater reductions in PAS.

## 2. Materials and Methods

This study was conducted as a retrospective study at the Department of Physical Medicine and Rehabilitation Istanbul Atlas University Medical Faculty Hospital, Istanbul, Türkiye. The study was designed in accordance with the Declaration of Helsinki and good clinical practice guidelines. It was approved by the Istanbul Atlas University Non-Interventional Scientific Research Ethics Committee (Ethics Committee approval date: 30 January 2026; number: E-22686390-050.99-90337). This study was reported in accordance with the STROBE (Strengthening the Reporting of Observational Studies in Epidemiology) guidelines.

### 2.1. Study Design and Data Source

This retrospective observational study was conducted using routinely collected clinical data of patients with dysphagia following ischemic stroke. All demographic, clinical, laboratory, and swallowing assessment data were obtained from patients’ electronic medical records. No additional diagnostic procedures, laboratory tests, or therapeutic interventions were performed specifically for the purposes of this study.

### 2.2. Participants

This retrospective study included adult patients (≥18 years) with a clinically and radiologically confirmed diagnosis of ischemic stroke who were followed at the study center between February 2023 and December 2025 and had documented post-stroke dysphagia requiring enteral feeding support, via either nasogastric tube or percutaneous endoscopic gastrostomy. All patients had undergone comprehensive clinical swallowing assessment and a videofluoroscopic swallowing study (VFSS) as part of routine dysphagia management and had available laboratory data corresponding to the defined assessment periods.

Patients were excluded if they had conditions known to significantly influence systemic inflammatory or nutritional biomarkers independent of stroke-related pathology. These included active infection, known malignancy, chronic inflammatory or autoimmune disorders, advanced hepatic dysfunction, advanced renal insufficiency, or other systemic diseases potentially affecting hematological or biochemical parameters. Patients with clinically documented active infection at the time of blood sampling were excluded to reduce confounding effects on inflammatory indices. However, due to the retrospective nature of the study, the presence of subclinical infections could not be completely ruled out.

Patients with incomplete clinical records, missing laboratory data required for index calculation, or unavailable swallowing assessment documentation were also excluded from the analysis. Eligible cases were identified using a consecutive sampling approach to minimize selection bias. No mortality was observed among the included patients during the study follow-up period.

### 2.3. Swallowing Assessment and Outcome Definitions

Swallowing function was evaluated using both functional and instrumental measures in accordance with routine institutional dysphagia management protocols.

#### 2.3.1. Functional Oral Intake Scale (FOIS)

Functional oral intake was evaluated using the Functional Oral Intake Scale (FOIS), a validated 7-point ordinal instrument developed to quantify the level of oral intake and tube dependence in individuals with dysphagia [9]. FOIS provides a structured classification of feeding status ranging from complete reliance on enteral nutrition to unrestricted oral intake, integrating both the route of nutrition and the degree of dietary modification required. Lower levels represent exclusive tube feeding or minimal oral intake, intermediate levels reflect progressive advancement to oral nutrition with texture modifications or preparation requirements, and higher levels indicate full oral intake with increasing dietary autonomy. FOIS was used in the study to analyze the functional implications of dysphagia, along with inflammatory and nutritional parameters [13].

In this study, FOIS scores at baseline (T0) and follow-up (T1) were retrospectively extracted from documented clinical swallowing evaluations recorded in patient charts as part of routine dysphagia management. The same institutional assessment framework was used across time points, ensuring consistency in clinical classification. FOIS was selected as the primary functional outcome measure because it reflects real-life feeding performance and nutritional independence rather than isolated physiological swallowing parameters. For longitudinal analyses, change scores (ΔFOIS = T1 − T0) were calculated to characterize recovery trajectories. Although FOIS is an ordinal scale, it was treated as a quasi-continuous variable in regression analyses, consistent with common practice in dysphagia outcome research, given its ordered structure and sensitivity to clinically meaningful functional change.

#### 2.3.2. Penetration–Aspiration Scale (PAS)

Airway invasion severity was evaluated using the Penetration–Aspiration Scale (PAS), an 8-point ordinal instrument developed to objectively quantify the depth of airway entry and the effectiveness of the patient’s protective response during videofluoroscopic swallowing studies (VFSSs) [10]. The scale provides a standardized framework for grading airway invasion events, ranging from the absence of penetration or aspiration to material passing below the vocal folds with an ineffective or absent protective response. The inter-rater reliability and clinical applicability of the PAS have been found to be high in both adult and pediatric populations. In validity and reliability studies conducted in Turkey, very high intraclass correlation coefficients (ICC > 0.95) have been reported across different consistencies and age groups. Due to these characteristics, PAS can be reliably used in retrospective studies based on existing videofluoroscopic recordings [14].

Lower scores indicate preserved airway protection, whereas higher scores reflect increasing severity of penetration and aspiration. Specifically, scores in the mid-range correspond to penetration events in which material enters the airway above the vocal folds, while the highest scores represent aspiration events characterized by bolus entry below the vocal folds, with or without an appropriate cough response.

In the present study, PAS scores at baseline (T0) and follow-up (T1) were retrospectively extracted from documented VFSS reports recorded as part of routine clinical dysphagia evaluation. Scores were analyzed separately for liquid and soft consistencies to account for potential consistency-dependent differences in airway protection. PAS was selected as the primary instrumental outcome reflecting swallowing physiology and aspiration severity. For longitudinal analyses, change scores (ΔPAS = T1 − T0) were calculated to characterize recovery dynamics. Although PAS is ordinal in structure, it was treated as a quasi-continuous variable in regression analyses, consistent with common practice in swallowing research when evaluating change over time in clinically ordered scales.

#### 2.3.3. Timing of Assessments

Timing of assessments was defined according to routine clinical dysphagia management within the same hospitalization episode. Baseline (T0) corresponded to the initial swallowing evaluation performed during the enteral feeding period in the chronic post-stroke phase, whereas follow-up (T1) represented reassessment after completion of structured dysphagia rehabilitation under standard institutional care. Both FOIS and PAS scores were retrospectively extracted from documented clinical and VFSS reports recorded at these time points. Laboratory values used for index calculations were obtained from routine blood tests performed during the same clinical periods corresponding to T0 and T1 assessments to ensure temporal alignment between biochemical and swallowing evaluations. Longitudinal change scores were calculated as Δ = T1 − T0 for all clinical and laboratory parameters and were used to characterize recovery dynamics over the rehabilitation interval.

#### 2.3.4. Definition of Transition to Oral Feeding

Transition from enteral to oral feeding was operationally defined as achieving functional oral intake without tube dependency at follow-up assessment. In accordance with FOIS staging, this corresponded to a FOIS score of ≥4 at T1. Within the FOIS framework, levels 1–3 represent varying degrees of tube dependence, whereas levels 4 and above indicate total oral intake without reliance on enteral nutrition, even when dietary modifications are required [9]. Therefore, the threshold of FOIS ≥ 4 was selected to reflect clinically meaningful discontinuation of tube feeding and restoration of oral nutritional autonomy. This operational definition aligns with established dysphagia outcome research in which removal of enteral support constitutes a key functional milestone in post-stroke recovery.

### 2.4. Stroke Severity Assessment

Baseline neurological severity was quantified using the National Institutes of Health Stroke Scale (NIHSS), a standardized and widely accepted measure of stroke-related neurological impairment. Given the retrospective design of the study, NIHSS scores were reconstructed from documented neurological examination findings recorded in patient charts at the time of initial clinical evaluation. Score estimation followed the validated record-based reconstruction methodology described by Kasner et al. (1999), which has demonstrated acceptable inter-rater reliability and agreement with prospectively obtained NIHSS scores when derived from comprehensive medical documentation [15].

NIHSS was not used as an inclusion or exclusion criterion. Instead, it was incorporated as a covariate in multivariable analyses to adjust for baseline neurological burden and to account for the potential confounding effect of stroke severity on swallowing outcomes and nutritional recovery. This approach allowed the independent contribution of inflammatory and nutritional indices to be evaluated while controlling for underlying neurological impairment.

### 2.5. Laboratory Parameters and Index Calculations

Laboratory data were retrospectively extracted from routine complete blood count and serum albumin measurements recorded in the institutional electronic database. Absolute neutrophil, lymphocyte, monocyte, and platelet counts were used to derive composite inflammatory indices reflecting systemic immune–inflammatory status, which have been increasingly investigated as prognostic markers in cardiovascular and neurovascular conditions. The neutrophil-to-lymphocyte ratio (NLR) and platelet-to-lymphocyte ratio (PLR) were calculated as ratios of absolute cell counts, representing shifts between innate and adaptive immune components and thrombocytic activation [3,4]. The systemic immune–inflammation index (SII) was computed as platelet × neutrophil/lymphocyte, consistent with the formulation proposed by Hu et al. (2014), integrating inflammatory and thrombotic pathways into a single parameter [6]. The systemic inflammation response index (SIRI), defined as neutrophil × monocyte/lymphocyte, was calculated according to Qi et al. (2016), incorporating monocyte-mediated inflammatory signaling [5]. The pan-immune–inflammation value (PIV), calculated as neutrophil × platelet × monocyte/lymphocyte, was derived following the methodology described by Zhao et al. (2022) and reflects a broader composite immune burden [8]. Nutritional status was assessed using the prognostic nutritional index (PNI), calculated as 10 × serum albumin (g/dL) + 0.005 × total lymphocyte count (/mm^3^), in accordance with established prognostic formulations (Sun et al., 2014), thereby integrating protein status and immune competence into a single metric [7]. All laboratory parameters corresponded to routine blood samples obtained during the same clinical periods as swallowing assessments at baseline (T0) and follow-up (T1), ensuring temporal alignment between biochemical and clinical measures. Longitudinal changes were calculated as Δ = T1 − T0 and used in subsequent analyses to characterize dynamic inflammatory and nutritional trajectories.

### 2.6. Statistical Analysis

Statistical analyses were performed using IBM SPSS Statistics for Windows, Version 26.0 (IBM Corp., Armonk, NY, USA). Continuous variables were summarized using mean, standard deviation, median, minimum–maximum values, and 95% confidence intervals. Normality was evaluated using skewness and kurtosis coefficients, histograms, and Q–Q plots. Non-parametric methods were applied when normality assumptions were not met.

Pre-therapy measurements were defined as T0 and post-therapy measurements as T1. Change scores were calculated as Δ = T1 − T0. Within-group comparisons between T0 and T1 were performed using the Wilcoxon signed-rank test. Comparisons between patients who transitioned to oral feeding and those who did not were performed using the Mann–Whitney U test.

Derived inflammatory and pan-nutritional indices (NLR, PLR, SII, SIRI, PNI, and PIV) were calculated according to established formulas. Values at T0 and T1 and their corresponding change scores (Δ) were included in the analyses.

Associations between changes in indices and changes in swallowing function (ΔFOIS, ΔPAS–Liquid, and ΔPAS–Soft) were examined using Spearman’s rank correlation analysis.

Indices showing significant correlations were further evaluated using multivariable linear regression analyses. To reduce overfitting and multicollinearity, each regression model included only one inflammatory or pan-nutritional index at a time. All linear regression models were adjusted for baseline stroke severity (NIHSS), age, and time since stroke (weeks). Results were reported as unstandardized β coefficients with 95% confidence intervals and *p* values.

To identify predictors of transition from enteral to oral feeding, multivariable logistic regression analysis was performed. The dependent variable was defined as post-therapy transition to oral feeding (1 = achieved, 0 = not achieved). The logistic model included baseline NIHSS and changes in inflammatory and pan-nutritional indices (ΔPNI, ΔNLR, ΔPLR, ΔSII, ΔSIRI, and ΔPIV). Age and time since stroke were not included in the logistic model to limit the number of predictors relative to the number of transition events and to reduce the risk of overfitting. Given the number of transition events (n = 48), model complexity was kept parsimonious. Odds ratios (ORs) with 95% confidence intervals were reported. Model fit was assessed using the likelihood ratio chi-square test, Nagelkerke R^2^, and receiver operating characteristic (ROC) curve analysis (AUC). Multicollinearity among predictors was assessed using variance inflation factor (VIF). All predictors showed low levels of collinearity, with VIF values indicating no evidence of problematic multicollinearity, even under conservative thresholds. This suggests that shared components among indices (e.g., lymphocyte count) did not substantially affect the stability of the regression models.

A post hoc evaluation of sample size adequacy was performed using G*Power software (Version 3.1.9.6; Heinrich Heine University, Düsseldorf, Germany) based on observed effect sizes. The available sample size and number of events were considered sufficient for the planned analyses.

## 3. Results

In this study, clinical and biochemical data obtained during the therapy process were presented for patients diagnosed with chronic dysphagia following ischemic stroke. Changes in laboratory parameters, inflammatory and pan-nutritional indices, and swallowing-related clinical scales were evaluated before and after therapy. Patients were additionally compared according to their transition status from enteral to oral feeding. The demographic and clinical characteristics of the study population are presented in Table 1.

According to Table 1, the study cohort consisted predominantly of older adults, with a balanced distribution between female and male participants. The majority of patients had experienced a middle cerebral artery (MCA) infarction, and a substantial proportion were receiving percutaneous endoscopic gastrostomy (PEG) feeding at baseline. The mean time since stroke was approximately one year.

Changes in swallowing-related clinical scales before and after therapy are presented in Table 2. Regarding swallowing-related outcomes, functional oral intake improved significantly, as evidenced by increased FOIS scores (*p* < 0.001), while aspiration severity decreased for both soft and liquid consistencies, reflected by significant reductions in PAS scores (both *p* < 0.001). Although albumin and total protein levels showed an upward trend after therapy, these changes did not reach statistical significance.

Following the observed improvements in swallowing outcomes, derived inflammatory and composite indices were evaluated to further characterize systemic inflammatory burden. NLR, PLR, SII, SIRI, PNI, and PIV were calculated at baseline and post-therapy, and comparisons are presented in Table 3. Significant post-therapy changes were observed in PLR, SII, SIRI, and PIV (all *p* < 0.05), although the direction of change differed across indices, with some increasing and others decreasing over time.

To further evaluate whether changes in inflammatory and pan-nutritional indices were associated with oral feeding transition, differences between post-therapy and pre-therapy measurements (Δ = T1 − T0) were calculated and compared between the two groups. These analyses are presented in Table 4. According to Table 4, change scores in inflammatory and pan-nutritional indices (ΔNLR, ΔPLR, ΔSII, ΔSIRI, ΔPNI, and ΔPIV) did not differ significantly between patients who did and did not transition to oral feeding (all *p* > 0.05). Although minor numerical differences were observed between groups, these findings suggest that changes in these indices alone are not sufficient to discriminate oral feeding transition at the group level and warrant further evaluation using correlation and multivariable analyses.

To examine whether changes in inflammatory and pan-nutritional status were associated with clinical changes in swallowing function, correlations were analyzed between change scores (Δ) of the derived indices and changes in functional oral intake (ΔFOIS) as well as changes in aspiration severity (ΔPAS–Liquid and ΔPAS–Soft). After multiple-comparison correction, only the most robust associations (primarily ΔPNI and ΔSIRI) remained statistically significant. These analyses, performed to determine the direction and strength of the relationships between biochemical and composite index changes and swallowing outcomes, are presented in Table 5. In Table 5, changes in inflammatory indices (ΔNLR, ΔPLR, ΔSII, ΔSIRI, and ΔPIV) were associated with less improvement in functional oral intake (ΔFOIS) and greater aspiration severity (ΔPAS), with ΔSIRI and ΔSII showing the most consistent significant correlations. ΔPNI was positively correlated with ΔFOIS and negatively correlated with both ΔPAS measures, indicating that changes in nutritional status are linked to better oral intake and reduced aspiration.

Indices that showed significant correlations with changes in swallowing outcomes were entered into multivariable regression models to assess their independent effects. To reduce overfitting and multicollinearity, each model included only one index and was adjusted for baseline NIHSS, time since stroke (weeks), and age. Separate models were constructed for ΔFOIS (Table 6), ΔPAS–Liquid (Table 7), and ΔPAS–Soft (Table 8). In Table 6, ΔPNI emerged as the strongest independent predictor of improvement in functional oral intake (ΔFOIS; B = 0.38, *p* < 0.001). In contrast, changes in inflammatory indices (ΔSIRI, ΔSII, ΔPLR, and ΔPIV) were independently associated with lower ΔFOIS. In Table 7, ΔPNI was independently associated with a reduction in liquid aspiration severity (ΔPAS–Liquid; B = −0.35, *p* = 0.001), whereas changes in inflammatory indices (ΔSIRI, ΔSII, ΔPLR, and ΔPIV) were independently associated with worse aspiration. In Table 8, ΔPNI was independently associated with a reduction in soft-consistency aspiration severity (ΔPAS–Soft; B = −0.29, *p* = 0.006), whereas changes in inflammatory indices (ΔSIRI, ΔSII, and ΔPIV) were independently associated with higher aspiration severity.

A multivariable logistic regression analysis was performed to identify predictors of post-therapy transition from enteral to oral feeding. A single multivariable logistic regression model was constructed including baseline NIHSS score and changes in all inflammatory and pan-nutritional indices (ΔPNI, ΔNLR, ΔPLR, ΔSII, ΔSIRI, and ΔPIV). Model fit was evaluated using the likelihood ratio chi-square test, explanatory power using Nagelkerke R^2^, and discriminative performance using receiver operating characteristic (ROC) curve analysis (Table 9).

To address concerns regarding potential overfitting, a reduced logistic regression model including only NIHSS and ΔPNI was constructed. The overall model was statistically significant (likelihood ratio *p* = 0.011) and demonstrated acceptable discriminative ability (AUC = 0.697). In this model, NIHSS remained a significant independent predictor of oral feeding transition (OR = 0.79, 95% CI: 0.67–0.93, *p* = 0.005), indicating that higher stroke severity was associated with a lower likelihood of transition to oral feeding. In contrast, ΔPNI was not independently associated with the outcome (OR = 1.02, 95% CI: 0.93–1.12, *p* = 0.637). The model explained a modest proportion of variance (Nagelkerke R^2^ = 0.149), supporting the primary role of clinical severity (NIHSS) over biomarker changes in predicting oral feeding transition (Table 10).

ROC analysis of the full multivariable model demonstrated good discriminative ability in predicting the post-therapy transition to oral feeding (AUC = 0.81, 95% CI: 0.70–0.91). At the optimal probability cut-off of 0.61, the sensitivity and specificity were 72% and 83%, respectively (Table 11).

**Figure 1:**
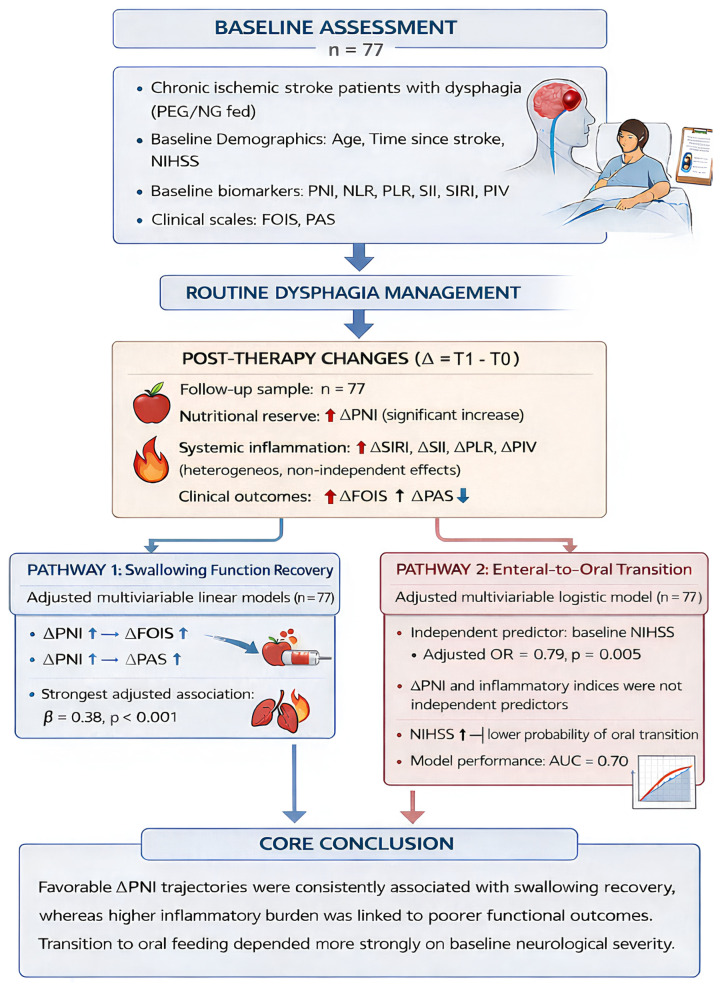
Conceptual framework of the study and main findings.

To synthesize the complex interactions between baseline clinical characteristics, biomarker dynamics, and swallowing rehabilitation outcomes, an integrated algorithmic framework of the study findings is presented in Figure 1. This comprehensive flowchart illustrates two distinct clinical pathways: the continuous recovery of swallowing function (driven independently by changes in nutritional status and blunted by systemic inflammation) and the categorical transition to oral feeding (primarily dictated by baseline neurological severity).

The flowchart illustrates the independent and relative associations of composite inflammatory indices (NLR, PLR, SII, SIRI, PIV) and the prognostic nutritional index (PNI) with dysphagia rehabilitation outcomes. Changes in nutritional status (ΔPNI) were independently associated with greater functional oral intake (ΔFOIS) and reduced aspiration severity (ΔPAS), whereas unfavorable changes in systemic inflammatory indices were associated with less favorable swallowing outcomes. In contrast, the transition from enteral to oral feeding was not associated with biomarker dynamics but was independently predicted by baseline neurological severity (NIHSS), with the multivariable model demonstrating good discriminative performance (AUC = 0.81). Δ indicates change between post-therapy (T1) and pre-therapy (T0). Arrows indicate the direction of relationships between variables and outcomes.

## 4. Discussion

The present study suggests that changes in nutritional reserve and systemic inflammatory burden are differentially associated with rehabilitation outcomes in chronic post-stroke dysphagia. Among the evaluated biomarkers, ΔPNI showed the most consistent association with changes in functional oral intake and aspiration-related outcomes, even after adjustment for age, time since stroke, and baseline neurological severity. In contrast, changes in composite inflammatory indices, particularly ΔSIRI, ΔSII, ΔPLR, and ΔPIV, were associated with less favorable swallowing trajectories. These findings should, however, be interpreted with caution. Given the retrospective design, the observational timing of the assessments, and the multiple statistical comparisons performed, the identified associations should not be interpreted as evidence of causality. In particular, changes in PNI may reflect overall clinical recovery occurring in parallel with swallowing improvement, and better swallowing itself may have contributed to improved nutritional intake, serum albumin, and lymphocyte-related indices. Accordingly, ΔPNI may be more appropriately interpreted as a pragmatic biomarker of systemic recovery and rehabilitation responsiveness rather than as an isolated mechanistic driver of swallowing recovery.

These findings are broadly consistent with the contemporary literature indicating that post-stroke dysphagia should not be viewed solely as a direct consequence of focal neurological injury, but rather within a broader framework of systemic vulnerability shaped by inflammation, immune dysregulation, and nutritional reserve [12,16]. Recent reviews have emphasized that malnutrition, aspiration risk, and systemic medical complications are closely intertwined in the clinical trajectory of dysphagic stroke patients and that dysphagia management should therefore include nutritional monitoring and supportive intervention as part of comprehensive care pathways [17]. Within this framework, the association between dynamic changes in PNI and better swallowing outcomes may reflect a clinically relevant signal of recovery readiness or overall physiological resilience.

### 4.1. Feeding Route Transition Versus Physiological Swallowing Recovery

In line with the primary aim of the study, we initially hypothesized that dynamic inflammatory and nutritional markers might independently relate not only to continuous swallowing improvement, but also to the binary transition from enteral to oral feeding. However, after multivariable adjustment, baseline neurological severity remained the only independent determinant of feeding route transition. This divergence is clinically meaningful and reinforces the need to distinguish threshold-based feeding decisions from physiological swallowing recovery. In everyday rehabilitation practice, the decision to initiate or advance oral feeding is rarely based on instrumental swallowing performance alone; rather, it reflects a broader judgment that integrates alertness, cooperation, fatigue, respiratory stability, infection risk, overall medical condition, and perceived safety of oral intake [12,18,19]. Accordingly, the absence of an independent association between biomarker dynamics and feeding route transition should not be interpreted as evidence that these markers are clinically irrelevant. Instead, our findings suggest that inflammatory and nutritional trajectories may be more informative for understanding the pace and quality of functional swallowing recovery, whereas the enteral-to-oral transition appears to remain primarily governed by neurological severity and overall clinical stability. This interpretation is also compatible with recent evidence suggesting that feeding restrictions and prolonged nil per os practices may not uniformly reduce pulmonary risk in post-stroke dysphagia and may, in selected patient groups, even accompany higher pneumonia burden [20]. Such observations further support the view that feeding route decisions are complex clinical judgments rather than direct reflections of a single biological signal.

### 4.2. Nutritional Reserve and Functional Dysphagia Recovery

Our secondary analyses showed that ΔPNI was the biomarker most consistently associated with improvement in both functional oral intake and aspiration-related outcomes. This pattern supports the interpretation that nutritional reserve is closely linked to the quality of dysphagia recovery; however, it should not be assumed that this relationship is unidirectional. In patients with chronic post-stroke dysphagia, better swallowing function may itself facilitate improved oral intake, greater dietary adequacy, and subsequent increases in serum albumin and lymphocyte-related indices. Accordingly, the association between ΔPNI and swallowing changes may reflect bidirectional or parallel recovery processes rather than a direct causal effect of nutritional status alone. Within this more cautious framework, ΔPNI may be understood as a dynamic clinical marker of physiological resilience and rehabilitation responsiveness. This interpretation is also compatible with previous evidence showing that nutritional support in stroke patients with dysphagia is associated with improved nutritional parameters, fewer complications, and better overall clinical recovery, although the causal pathway linking nutritional improvement to swallowing physiology remains difficult to disentangle in retrospective designs [21]. Moreover, because lymphocyte count contributes both to PNI and to the denominator of several composite inflammatory indices, part of the apparent divergence between improving PNI and worsening inflammatory ratios may reflect shared hematological dynamics rather than fully distinct biological mechanisms [22]. While previous studies support the broader prognostic relevance of PNI in dysphagia and stroke-related populations [23], the present findings suggest that ΔPNI may also serve as a sensitive marker of recovery trajectories for dysphagia-related outcomes measured by FOIS and PAS. Therefore, PNI may be better positioned as a monitoring biomarker reflecting qualitative changes in swallowing safety and efficiency, rather than as a standalone determinant of feeding route transition.

### 4.3. Systemic Inflammation as a Biological Constraint on Recovery

Conversely, the observation that changes in inflammatory indices were associated with less favorable swallowing outcomes may indicate that persistent systemic inflammatory burden accompanies a less efficient rehabilitation trajectory. This interpretation is consistent with prior stroke research showing that higher SII is associated with greater stroke severity and worse clinical outcomes in acute ischemic stroke [24], and that SIRI is independently associated with functional outcomes in severe stroke populations, including patients undergoing mechanical thrombectomy [25]. Although these studies are not dysphagia-specific, they support the broader plausibility that composite inflammatory burden may track a more fragile recovery state after stroke. Moreover, a recent study in ischemic stroke complicated by pulmonary infection demonstrated the prognostic relevance of SII-based indices, suggesting that inflammatory burden may be particularly consequential in clinical phenotypes in which respiratory complications are prominent [26]. Given the high frequency of aspiration and pulmonary complications in post-stroke dysphagia, indices such as SII and SIRI may therefore serve as accessible markers of clinical vulnerability in this population.

At the same time, the apparent increase in several CBC-derived inflammatory indices over the rehabilitation interval should be interpreted cautiously. These indices are highly sensitive to intercurrent systemic factors in post-stroke populations, and subclinical or intercurrent infections, including stroke associated pneumonia or urinary tract infections, may not be fully captured in retrospective records, even when patients with overt infection are excluded [12,18]. In addition, these indices may reflect complex immune trajectories during recovery, including stroke-induced immunodepression, medication effects, and lymphocyte dynamics, rather than a simple inflammation resolution process [16]. Therefore, increasing SII, SIRI, and PIV values during the rehabilitation period should not be interpreted as evidence against swallowing recovery. Rather, they may be better understood as practical biomarkers of systemic vulnerability that can coexist with functional gains.

Accordingly, although post-therapy changes in composite inflammatory indices did not independently predict the categorical transition to full oral feeding, higher or persistent inflammatory responses were independently associated with poorer functional trajectories, including lower gains in functional oral intake and greater residual aspiration severity. In this context, inflammatory indices may be more appropriately interpreted as adjunctive monitoring markers of recovery quality rather than direct mechanistic determinants of swallowing rehabilitation.

### 4.4. Clinical Implications

Collectively, our findings support a two-level clinical interpretation of post-stroke dysphagia recovery. While baseline neurological severity appears to guide threshold-based decisions such as the transition from enteral to oral feeding, dynamic changes in nutritional reserve and systemic inflammatory burden may help explain variation in the pace and quality of functional swallowing recovery. Rather than dictating feeding route decisions, these routine and cost-effective indices may serve as practical biomarkers for longitudinal monitoring and for identifying patients who appear more vulnerable to inflammation-associated recovery limitation. From a clinical perspective, these findings support a broader rehabilitation framework in which swallowing outcomes are interpreted not only through neurological severity, but also through the patient’s evolving nutritional and systemic inflammatory status.

### 4.5. Limitations

Several limitations should be considered when interpreting these findings. First, the retrospective single-center design and the exclusion of incomplete records limit both generalizability and causal inference. This may also have introduced selection bias by favoring patients who were clinically stable enough to complete repeated assessments, thereby potentially inflating observed recovery patterns. Second, CBC-derived inflammatory indices are not specific to stroke-related dysphagia and may have been influenced by unmeasured factors such as subclinical infection, medication use, and medical comorbidities, especially because systematic data on acute-phase reactants such as C-reactive protein and detailed clinical histories were not consistently available. Third, because direct body composition measures were not obtained, formal diagnosis of sarcopenia and objective quantification of muscle mass changes alongside ΔPNI were not possible. Fourth, the transition to oral feeding is partly shaped by institutional practice patterns and safety-based clinical decision making, which may weaken its association with individual biomarkers. Finally, lymphocyte count contributes both to PNI and to several composite inflammatory indices, which may partly amplify the apparent inverse relationship between nutritional and inflammatory trajectories. For this reason, these biomarker associations should be interpreted as complementary clinical signals rather than evidence of fully independent biological pathways. Future prospective multicenter studies with standardized longitudinal assessments are needed to validate these biomarker trajectories, better account for confounding factors, and determine whether targeted nutritional or anti-inflammatory strategies can improve dysphagia-related outcomes.

## 5. Conclusions

This retrospective study suggests that recovery in chronic post-stroke dysphagia may follow partly distinct clinical trajectories. ΔPNI was the biomarker most consistently associated with improvement in functional oral intake and aspiration-related outcomes. Because ΔPNI can be derived from routine laboratory data, it may represent a practical biomarker for longitudinal monitoring of clinically meaningful recovery. In contrast, higher or persistent inflammatory burden was associated with less favorable functional trajectories, whereas the transition from enteral to complete oral feeding appeared to remain more strongly related to baseline neurological severity than to isolated biomarker fluctuations. Overall, these findings support a multidimensional view of dysphagia rehabilitation in which neurological severity, nutritional reserve, and systemic inflammatory status should be interpreted together. Future prospective multicenter studies are needed to confirm these biomarker trajectories, clarify causal relationships, and determine whether targeted nutritional or anti-inflammatory strategies can improve rehabilitation outcomes.

## Figures and Tables

**Table 1:** Demographic and clinical characteristics of the participants.

Variable	Category	n	%
Age (years)	Mean ± SD	61.7 ± 14.9	-
Time since stroke (weeks)	Mean ± SD	46.0 ± 26.4	-
NIHSS	Mean ± SD	8.3 ± 4.2	-
Sex	Female (F)	39	50.6
	Male (M)	38	49.4
Feeding method (T0)	PEG	55	71.4
	NG	22	28.6
Oral transition	Oral transition	48	62.3
	No transition	29	37.7
Stroke type	MCA	53	68.8
	ACA	18	23.4
	PCA	6	7.8

PEG, percutaneous endoscopic gastrostomy; NG, nasogastric tube; MCA, middle cerebral artery; ACA, anterior cerebral artery; PCA, posterior cerebral artery.

**Table 2:** Comparison of clinical scales before and after therapy.

				Pre-Therapy			Post-Therapy				
Variable	n	Min	Max	Mean	SD	Min	Max	Mean	SD	Z	*p*
FOIS	77	1	3	1.87	0.85	3	7	5.51	0.97	−7.681	**<0.001**
PAS–Soft Consistency	77	5	7	5.80	0.80	1	3	1.69	0.71	−7.765	**<0.001**
PAS–Liquid	77	6	8	6.58	0.74	1	4	2.03	0.97	−7.713	**<0.001**

FOIS, Functional Oral Intake Scale; PAS, Penetration–Aspiration Scale. Statistical test: Wilcoxon signed-rank test. Bold values indicate statistically significant *p*-values (*p* < 0.05).

**Table 3:** Changes in derived inflammatory and pan-nutritional indices before and after therapy.

	Pre-Therapy						Post-Therapy							
	N	Min	Max	Mean	SD	Median (IQR)	N	Min	Max	Mean	SD	Median (IQR)	Z	*p*
NLR	77	0.01	4.74	0.47	0.55	0.32 (0.18–0.56)	77	0.43	37.12	4.69	6.00	2.85 (1.40–5.60)	−1.283	0.199
PLR	77	14.3	312.6	98.4	66.1	82.3 (52.1–128.7)	77	42.7	680.4	184.9	141.2	152.4 (92.5–238.6)	−2.041	**0.041**
SII	77	84.03	10,076.0	1341.5	1617.1	820.1 (432.8–1560.3)	77	143.29	4446.3	975.77	921.06	722.5 (447.7–1225.0)	−1.954	**0.049**
SIRI	77	0.02	3.41	0.42	0.63	0.21 (0.11–0.48)	77	0.11	9.74	2.31	2.14	1.65 (0.80–2.90)	−2.117	**0.034**
PNI	77	35.51	51.81	46.00	4.84	42.10 (39.20–44.90)	77	29.0	51.61	40.54	5.03	40.70 (37.80–43.50)	−1.890	0.059
PIV	77	0.01	42.6	6.14	8.93	3.10 (1.50–7.20)	77	0.12	286.4	58.7	61.9	34.5 (18.2–72.4)	−2.436	**0.015**

NLR, neutrophil-to-lymphocyte ratio; PLR, platelet-to-lymphocyte ratio; SII, systemic immune–inflammation index (platelet × neutrophil/lymphocyte); SIRI, systemic inflammation response index (neutrophil × monocyte/lymphocyte); PNI, prognostic nutritional index [10 × albumin (g/dL) + 0.005 × total lymphocyte count (/mm^3^)]; PIV, pan-immune–inflammation value [(neutrophil × platelet × monocyte)/lymphocyte]. Z and *p* values were calculated using the Wilcoxon signed-rank test. Bold values indicate statistically significant *p*-values (*p* < 0.05).

**Table 4:** Comparison of changes (Δ) in inflammatory and pan-nutritional indices between patients who did and did not transition to oral feeding.

	Patients Who Transitioned to Oral Feeding					Patients Who Did Not Transition to Oral Feeding						
ΔVariable	N	Min	Max	Mean	SS	N	Min	Max	Mean	SS	U	*p*
ΔNLR	48	−31.08	19.01	−0.39	6.75	29	−9.66	29.55	1.91	7.50	691.00	0.962
ΔPLR	48	−1052.56	340.60	−2.73	177.07	29	−206.87	688.75	30.99	180.00	752.50	0.556
ΔSII	48	−3393.70	7416.70	376.94	1632.95	29	−2463.94	7744.95	571.81	2351.12	712.50	0.866
ΔSIRI	48	−9.43	28.63	1.29	4.82	29	−11.16	32.21	1.91	8.40	702.50	0.950
ΔPNI	48	−18.40	10.09	−1.13	6.58	29	−7.95	4.90	−1.60	3.20	775.00	0.665
ΔPIV	48	−2377.39	8446.50	372.49	1430.62	29	−2372.22	8771.61	520.76	2205.86	714.50	0.850

Δ indicates change between post-therapy and pre-therapy measurements (Δ = T1 − T0). Continuous variables are presented as mean ± standard deviation (SD). Group comparisons were performed using the Mann–Whitney U test.

**Table 5:** Associations between changes in inflammatory and pan-nutritional indices and changes in swallowing function.

Variable	ΔFOIS		ΔPAS–Liquid		ΔPAS–Soft Consistency	
	r	*p*	r	*p*	r	*p*
ΔNLR	−0.21	0.048	0.19	0.082	0.14	0.204
ΔPLR	−0.28	**0.013**	0.24	**0.031**	0.18	0.096
ΔSII	−0.31	**0.006**	0.29	**0.011**	0.22	0.047
ΔSIRI	−0.34	**0.003**	0.33	**0.004**	0.26	**0.021**
ΔPNI	0.41	**<0.001**	−0.38	**0.001**	−0.31	**0.006**
ΔPIV	−0.27	**0.017**	0.25	**0.026**	0.21	0.049

Δ = post-therapy (T1) − pre-therapy (T0). A *p* value < 0.05 was considered statistically significant. Associations were assessed using Spearman’s rank correlation. Benjamini–Hochberg correction was applied to control for multiple comparisons; only associations that remained significant after correction are highlighted in bold.

**Table 6:** Multivariable linear regression models for changes in functional oral intake (ΔFOIS).

Model	Independent Variable	B	95% CI	*p*
Model 1	ΔPNI	0.38	0.21, 0.55	**<0.001**
Model 2	ΔSIRI	−0.26	−0.43, −0.08	**0.004**
Model 3	ΔSII	−0.23	−0.40, −0.06	**0.009**
Model 4	ΔPLR	−0.21	−0.38, −0.04	**0.015**
Model 5	ΔPIV	−0.19	−0.35, −0.02	**0.028**

Δ = T1 − T0. Separate multivariable linear regression analyses were performed for each inflammatory or pan-nutritional index. Each model included baseline NIHSS score, age, and time since stroke (weeks) as covariates, in addition to the respective index. Only the coefficients of the indices of interest are presented. B values denote unstandardized regression coefficients with 95% confidence intervals (CI). A two-sided *p* value < 0.05 was considered statistically significant. Bold values indicate statistically significant *p*-values (*p* < 0.05).

**Table 7:** Multivariable linear regression models for changes in liquid aspiration severity (ΔPAS–Liquid).

Model	Independent Variable	B	95% CI	*p*
Model 1	ΔPNI	−0.35	−0.52, −0.18	**0.001**
Model 2	ΔSIRI	0.31	0.14, 0.48	**0.002**
Model 3	ΔSII	0.27	0.09, 0.45	0.004
Model 4	ΔPLR	0.22	0.04, 0.40	0.018
Model 5	ΔPIV	0.20	0.02, 0.38	0.031

Δ = T1 − T0. Separate multivariable linear regression analyses were performed for each inflammatory or pan-nutritional index. Each model included baseline NIHSS score, age, and time since stroke (weeks) as covariates, in addition to the respective index. Only the coefficients of the indices of interest are presented. B values denote unstandardized regression coefficients with 95% confidence intervals (CI). A two-sided *p* value < 0.05 was considered statistically significant. Bold values indicate statistically significant *p*-values (*p* < 0.05).

**Table 8:** Multivariable linear regression models for changes in soft-consistency aspiration severity (ΔPAS–Soft).

Model	Independent Variable	B	95% CI	*p*
Model 1	ΔPNI	−0.29	−0.46, −0.12	**0.006**
Model 2	ΔSIRI	0.24	0.06, 0.42	0.011
Model 3	ΔSII	0.21	0.02, 0.40	0.028
Model 4	ΔPIV	0.19	0.01, 0.37	0.041

Δ = T1 − T0. Separate multivariable linear regression analyses were performed for each inflammatory or pan-nutritional index. Each model included baseline NIHSS score, age, and time since stroke (weeks) as covariates, in addition to the respective index. Only the coefficients of the indices of interest are presented. B values denote unstandardized regression coefficients with 95% confidence intervals (CI). A two-sided *p* value < 0.05 was considered statistically significant. Bold values indicate statistically significant *p*-values (*p* < 0.05).

**Table 9:** Predictors of post-therapy transition from enteral to oral feeding.

Variable	OR	%95 CI	*p*
NIHSS	0.72	0.59, 0.89	**0.002**
ΔPNI	1.01	0.98, 1.04	0.412
ΔNLR	0.91	0.71, 1.17	0.471
ΔPLR	0.99	0.98, 1.01	0.622
ΔSII	0.88	0.64, 1.20	0.418
ΔSIRI	1.02	0.77, 1.36	0.884
ΔPIV	0.97	0.71, 1.33	0.861

OR, odds ratio; CI, confidence interval; NIHSS, National Institutes of Health Stroke Scale; Δ indicates change between post-therapy and pre-therapy measurements (Δ = T1 − T0). Bold values indicate statistically significant *p*-values (*p* < 0.05).

**Table 10:** Reduced logistic regression model for oral feeding transition.

Variable	β	SE	95% CI	*p*
NIHSS	−0.238	0.086	0.79 (0.67–0.93)	**0.005**
ΔPNI	0.021	0.045	1.02 (0.93–1.12)	0.637

NIHSS, National Institutes of Health Stroke Scale; Δ indicates change between post-therapy and pre-therapy measurements (Δ = T1 − T0). Bold values indicate statistically significant *p*-values (*p* < 0.05).

**Table 11:** Discriminative performance of the final model (ROC analysis).

Parameters	Value
AUC (ROC)	0.81
95% Confidence interval	0.70–0.91
Optimal cut-off (probability)	0.61
Sensitivity (%)	72
Specificity (%)	83

ROC, receiver operating characteristic; AUC, area under the curve; CI, confidence interval.

## Data Availability

The data presented in this study are available on request from the corresponding author.

## Abbreviations

The following abbreviations are used in this manuscript:

ACA	Anterior cerebral artery
AUC	Area under the curve
CI	Confidence interval
FOIS	Functional Oral Intake Scale
MCA	Middle cerebral artery
NG	Nasogastric tube
NIHSS	National Institutes of Health Stroke Scale
NLR	Neutrophil-to-lymphocyte ratio
PAS	Penetration–Aspiration Scale
PEG	Percutaneous endoscopic gastrostomy
PLR	Platelet-to-lymphocyte ratio
PNI	Prognostic nutritional index
PIV	Pan-immune–inflammation value
PCA	Posterior cerebral artery
SII	Systemic immune–inflammation index
SIRI	Systemic inflammation response index

## References

[1] Anrather J., Iadecola C. (2016). Inflammation and Stroke: An Overview. Neurotherapeutics.

[2] Correia M.I.T.D., Waitzberg D.L. (2003). The Impact of Malnutrition on Morbidity, Mortality, Length of Hospital Stay and Costs Evaluated through a Multivariate Model Analysis. Clin. Nutr..

[3] Siqueira J.M., Soares J.D.P., Borges T.C., Gomes T.L.N., Pimentel G.D. (2021). High Neutrophil-to-Lymphocyte Ratio Is Associated with Nutritional Risk in Hospitalized Patients. Sci. Rep..

[4] Duan H., Zhang J., Wang P., Zhang J., Jiang J. (2023). Association between Nutritional Status and Platelet-to-Lymphocyte Ratio in Patients with Hepatocellular Carcinoma Undergoing Transcatheter Arterial Chemoembolization. Nutr. Hosp..

[5] Qi Q., Zhuang L., Shen Y., Geng Y., Yu S., Chen H., Shi Y. (2016). A Novel Systemic Inflammation Response Index (SIRI) Predicts the Survival of Patients with Pancreatic Cancer after Chemotherapy. Cancer.

[6] Hu B., Yang X.R., Xu Y., Sun Y.F., Sun C., Guo W., Fan J. (2014). Systemic Immune-Inflammation Index Predicts Prognosis of Patients after Curative Resection for Hepatocellular Carcinoma. Clin. Cancer Res..

[7] Sun K., Chen S., Xu J., Li G., He Y. (2014). The Prognostic Significance of the Prognostic Nutritional Index in Cancer: A Systematic Review and Meta-Analysis. J. Cancer Res. Clin. Oncol..

[8] Zhao H., Chen X., Zhang W., Cheng D., Lu Y., Wang C., Huang Y. (2022). Pan-Immune-Inflammation Value Is Associated with the Clinical Stage of Colorectal Cancer. Front. Surg..

[9] Crary M.A., Mann G.D.C., Groher M.E. (2005). Initial Psychometric Assessment of a Functional Oral Intake Scale for Dysphagia in Stroke Patients. Arch. Phys. Med. Rehabil..

[10] Rosenbek J.C., Robbins J.A., Roecker E.B., Coyle J.L., Wood J.L. (1996). A Penetration–Aspiration Scale. Dysphagia.

[11] Adams H.P., Davis P.H., Leira E.C., Chang K.C., Bendixen B.H., Clarke W.R., Woolson R.F., Hansen M.D. (1999). Baseline NIH Stroke Scale Score Strongly Predicts Outcome after Stroke: A Report of the Trial of Org 10172 in Acute Stroke Treatment (TOAST). Neurology.

[12] Martino R., Foley N., Bhogal S., Diamant N., Speechley M., Teasell R. (2005). Dysphagia after Stroke: Incidence, Diagnosis, and Pulmonary Complications. Stroke.

[13] Salassa J.R. (1999). A Functional Outcome Swallowing Scale for Staging Oropharyngeal Dysphagia. Dig. Dis..

[14] Karaduman A., Serel S., Ünlüer Ö., Demir N. (2012). Penetrasyon Aspirasyon Skalası: Kişiler Arası Güvenirlik Çalışması. Fizyoter. Rehabil..

[15] Kasner S.E., Chalela J.A., Luciano J.M., Cucchiara B.L., Raps E.C., McGarvey M.L., Conroy M.B., Localio A.R. (1999). Reliability and Validity of Estimating the NIH Stroke Scale Score from Medical Records. Stroke.

[16] Meisel C., Schwab J.M., Prass K., Meisel A., Dirnagl U. (2005). Central Nervous System Injury-Induced Immune Deficiency Syndrome. Nat. Rev. Neurosci..

[17] Labeit B., Michou E., Trapl-Grundschober M., Suntrup-Krueger S., Muhle P., Bath P.M., Dziewas R. (2024). Dysphagia after Stroke: Research Advances in Treatment Interventions. Lancet Neurol..

[18] Hoffmann S., Harms H., Ulm L., Nabavi D.G., Mackert B.M., Schmehl I., Jungehulsing G.J., Montaner J., Bustamante A., Hermans M. (2017). Stroke-Induced Immunodepression and Dysphagia Independently Predict Stroke-Associated Pneumonia—The PREDICT Study. J. Cereb. Blood Flow Metab..

[19] Singer P., Blaser A.R., Berger M.M., Alhazzani W., Calder P.C., Casaer M.P., Hiesmayr M., Mayer K., Montejo J.C., Pichard C. (2019). ESPEN Guideline on Clinical Nutrition in the Intensive Care Unit. Clin. Nutr..

[20] Ihrke M., Meisel A., Nelde A., Neumann K., Mürbe D., Voß L.J., Caffier P.P. (2025). The Crux of NPO Paradox Revealed by Increased Pneumonia Incidence in Post-Stroke Dysphagia Patients with Dietary Restrictions. Sci. Rep..

[21] Zhu C., Lu X., Zhang Y., Miao W., Huang H., Lu Q. (2025). The Impact of Early Nutritional Intervention on Nutritional Status, Neurological Deficit, and Complications in Stroke Patients with Dysphagia: A Meta-Analysis. J. Hum. Nutr. Diet..

[22] Ustaalioğlu İ., Umaç G.A. (2024). The role of the prognostic nutritional index in predicting mortality in stroke patients. Rev. Assoc. Medica Bras..

[23] Pei R., Wang D. (2025). Prognostic Nutritional Index Negatively Associated with Mortality in Older Japanese Patients with Dysphagia. Front. Nutr..

[24] Rao Z., Zhang Y., Zhu C. (2025). Association of Systemic Immune-Inflammation Index with Severity in Acute Ischemic Stroke Patients: A Cross-Sectional Study. Front. Neurol..

[25] Wu W., Zhang Y.P., Qu X.G., Zhang Z.H. (2024). Association of the Systemic Inflammation Response Index with Functional Outcome in Acute Large Vessel Occlusion Stroke Patients Receiving Mechanical Thrombectomy. J. Inflamm. Res..

[26] Chen D., Bin M., Kang L., Chen L., Hu H. (2025). Prognostic Evaluation of Ischemic Stroke Complicated by Pulmonary Infection Using SII and CALLY Indices. Am. J. Transl. Res..

